# Necrotizing Soft Tissue Infection Secondary to Spinal Hardware Malfunction: A Case Report on Surgical Debridement

**DOI:** 10.7759/cureus.85917

**Published:** 2025-06-13

**Authors:** Patrick D Plummer, Landry Umbu, Penelope Mashburn, Ryan Debiec

**Affiliations:** 1 Department of Surgery, Western Reserve Health Education, Northeast Ohio Medical University (NEOMED), Warren, USA; 2 Department of Surgery, Trumbull Regional Medical Center, Warren, USA

**Keywords:** emphysema subcutaneous, necrotizing fasciitis (nf), necrotizing soft tissue infection (nsti), spinal hardware, urgent debridement

## Abstract

Necrotizing soft tissue infections (NSTIs) are characterized by “flesh-eating bacteria” that are rapidly progressive and require immediate diagnosis and aggressive surgical debridement along with broad-spectrum antibiotics. Common pathogens, such as *Streptococcus pyogenes*, *Staphylococcus aureus*, *Enterococcus coli*, and Clostridium, are the usual culprits of this disease. While NSTIs have a relatively low incidence, they are associated with higher rates of morbidity and mortality. Due to the aggressive nature of the pathogen, NSTI can cause widespread necrosis of soft tissue and muscle, leading to extensive surgical intervention and patient disfigurement. The patient population that is most susceptible to NSTI includes the elderly, immunocompromised, and diabetics. In this case report, the patient is a 54-year-old woman with a past medical history of type 2 diabetes mellitus, hepatitis C, and an extensive spinal surgery with malfunctioning spinal hardware who developed an NSTI while in the intensive care unit.

## Introduction

Necrotizing soft tissue infection (NSTI) is a rare but potentially lethal infection with a population incidence rate of four per 100,000 per year [1,2]. Patients between 40 and 60 years of age are at the highest risk of developing NSTI, with a higher prevalence in men. Individuals living with certain comorbidities are at higher risk for developing NSTI compared to the general population. Comorbidities associated with NSTI include diabetes mellitus (22%-59%), obesity (17%-31%), cardiovascular disease (9%-45%), and immunosuppressive illness (4%-30%) [1,2]. The recurrence rate of patients previously treated for NSTI ranges from 4% to 12%.

NSTI is characterized by infiltration of bacteria into the subcutaneous tissue, superficial fascia, and muscle, causing widespread necrosis [1,2]. NSTI serves as an umbrella term for a wide range of pathogens that can cause necrosis of soft tissue and muscle. These types of infections can occur in various anatomical regions within the body, including but not limited to the orbital, cervical, upper and lower extremities, thoracoabdominal, and perineal/genital regions [1,2]. Usually, a breakdown in the skin barrier can introduce these harmful bacteria into the body. In 10%-30% of NSTI cases, local trauma was indicated as the portal of entry [1-3]. Some examples include minor skin abrasions, postoperative surgical wounds, and insect bites, which were all noted as potential sites of entry. The incidence rate of surgical site infections (SSIs) secondary to spinal implants varies from 2.45% to 20% due to the inability to completely remove hardware implants without causing spinal destabilization [4,5].

The pathogens that cause NSTI can be classified into three distinctive categories. Type I is polymicrobial, which is a mixture of anaerobes and aerobes; this type of NSTI accounts for 70%-80% of cases [1-3]. Type II contributes approximately 20%-30%, which is monomicrobial and includes Group A Beta-hemolytic streptococcus and *Staphylococcus aureus*, which produce exotoxins that destroy the surrounding tissue [1-3]. Finally, Type III is associated with marine-related organisms such as Vibrio species [3]. This type of NSTI is mostly prevalent in Asian countries and is acquired via seafood ingestion or contaminated water in open wounds. Necrotizing fasciitis (NF) is a type of NSTI that is propagated along the deep fascia layer, causing severe necrosis to surrounding tissue [3].

While clinical suspicion of NSTI is enough to administer treatment, additional diagnostic modalities can be used to support the diagnosis. Stable patients should undergo a computed tomography (CT) scan with intravenous contrast, which can help reveal subcutaneous emphysema or fat stranding, lack of enhancement in underlying fascia, and subcutaneous fluid collection. While subcutaneous emphysema is a strong clinical sign of NF, it is only present in 30% of cases [3,4]. Other diagnostic imaging includes plain radiographs, ultrasonography, and MRI [3,4].

The Laboratory Risk Indicator for Necrotizing Fasciitis (LRINEC) score is a systematic approach designed to help identify a patient’s probability of developing an NSTI [1-4]. The score uses glucose, white blood cell count, hemoglobin, sodium, serum creatinine, and C-reactive protein to devise a score for the likelihood of developing an NSTI. A score >6 indicates a positive predictive value of 92% and a negative predictive value of 96% [3,4].

At the bedside, physical examination may show evidence of “dishwasher” fluid drainage, crepitus upon deep palpation of tissue, and a positive finger test [3,4]. When diagnosis remains unclear, you can perform a bedside cutdown under local anesthesia. The return of foul-smelling dishwater fluid will confirm your diagnosis. Treatment includes emergent surgical debridement along with broad-spectrum antibiotics and intensive care monitoring.

## Case presentation

The patient is a 54-year-old woman with a past medical history of uncontrolled diabetes mellitus, urinary tract infections, and spinal surgery with metal hardware implants brought by emergency medical services to the emergency department (ED) due to elevated glucose levels, altered mental status, and tachycardia. The patient’s family states she has not been checking her blood sugar levels regularly for the past couple of weeks. On admissions, the initial workup included complete blood count, comprehensive metabolic panel, arterial blood gas, lactic acid, and beta-hydroxybutyric, which suggested that the patient was in DKA (Tables 1-3).

**Table 1:** Comprehensive metabolic panel on admissions BUN: blood urea nitrogen

Comprehensive metabolic panel	Results	Reference ranges and units
Sodium	121	135-145 mmol/L
Potassium	3.4	3.5-4.5 mmol/L
Chloride	81	98-107 mmol/L
Anion gap	7	5-15 mmol/L
BUN	15	5-25 mmol/L
Creatinine	0.9	0.6-1.4 mmol/L
Glucose	672	<100 mmol/L
A1c	19.7	<6.5 mmol/L
Lactic acid	3.8	0.5-2 mmol/L
Beta-hydroxybutyric acid	6.57	0.6-1.5 mmol/L

**Table 2:** Arterial blood gas on admissions pCO_2_: partial pressure of carbon dioxide; pO_2_: partial pressure of oxygen; HCO_3_: bicarbonate

Arterial blood gas	Results	Reference ranges and units
pH	7.17	7.35-7.45
pCO_2_	16.7	32-45 mmHg
pO_2_	134	75-100 mmHg
HCO_3_	6	20-24 mmHg

**Table 3:** Complete blood count on admissions

Complete blood count	Results	Reference ranges and units
White blood cell	25.1	3.5-10.5 × 10^3^/uL
Red blood cell	5.18	3.80-5.40 × 10^6^/uL
Hemoglobin	11.9	12-15.5 g/dL
Hematocrit	38.5	35%-45%

**Figure 1:**
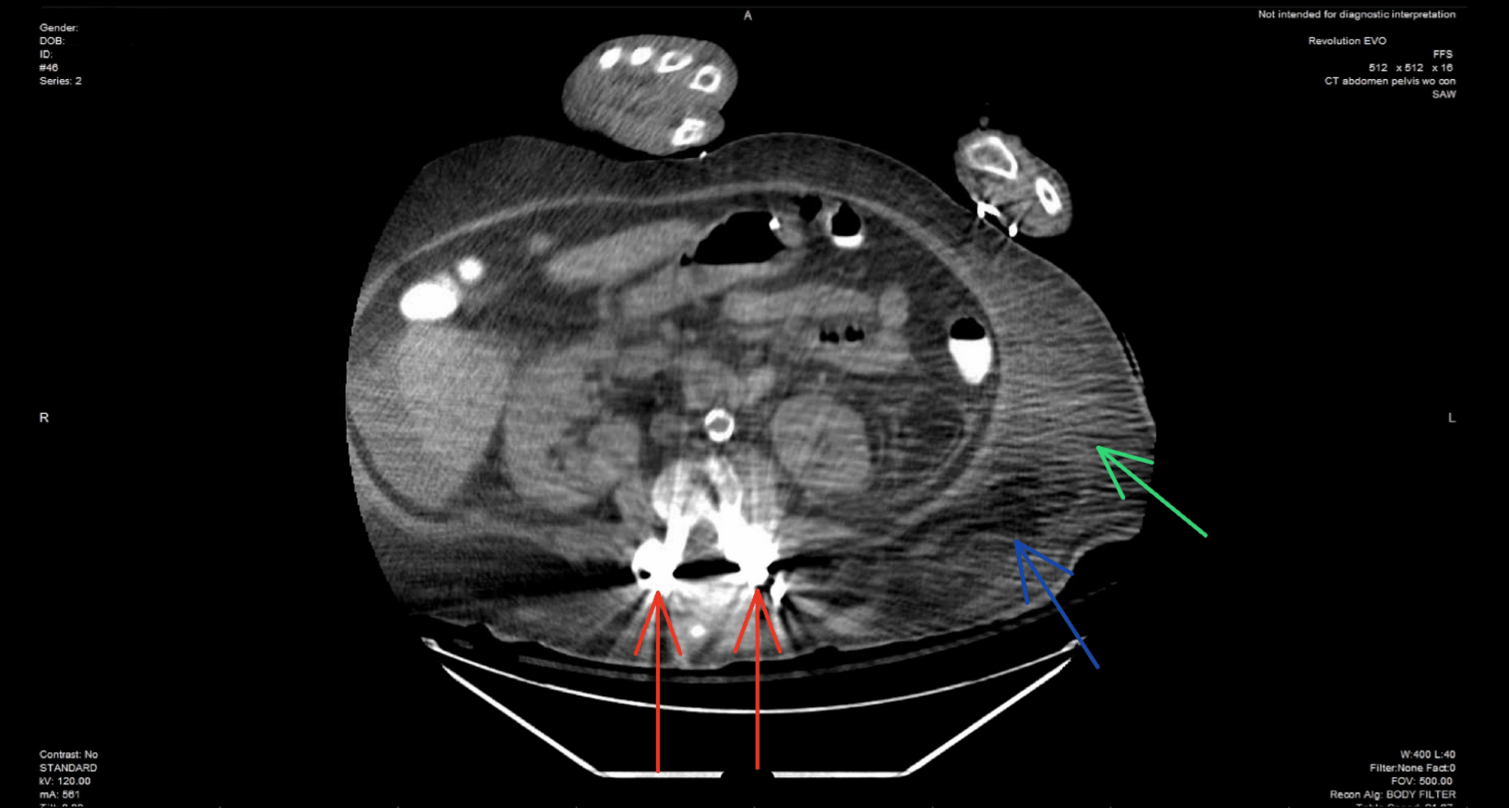
Axial view at the level of the spine, CT scan of the abdomen/pelvis shows spinal hardware implants (red arrows), subcutaneous emphysema (blue arrow), and subcutaneous fat standing (green arrow) CT: computed tomography

**Figure 2:**
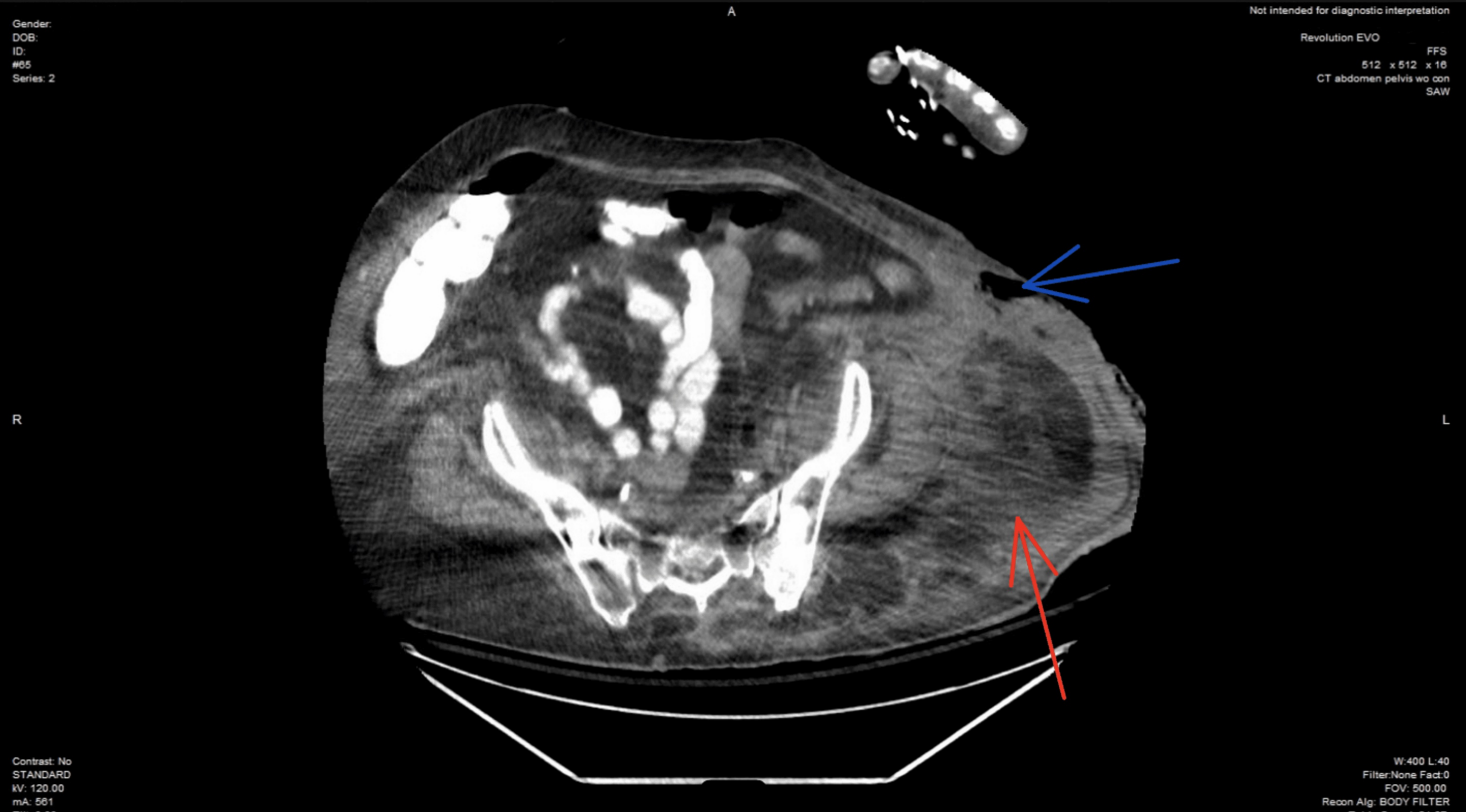
Axial view at the level of the sacrum, CT scan of the abdomen/pelvis shows subcutaneous emphysema (blue arrow), and subcutaneous fat standing (red arrow) CT: computed tomography

**Figure 3:**
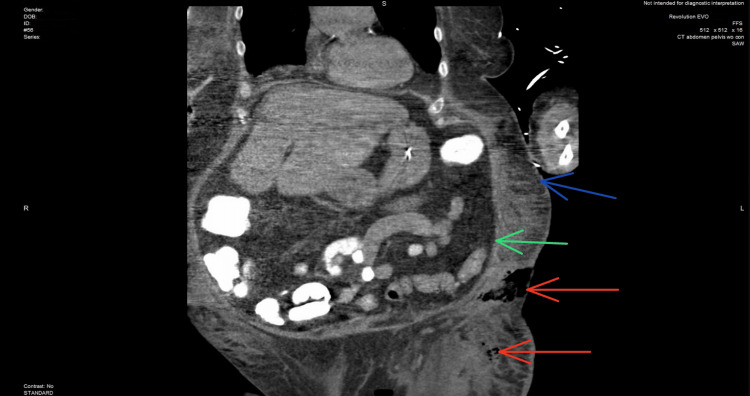
Coronal view shows subcutaneous emphysema in the lower lateral abdomen and superior thigh (red arrows), subcutaneous fat stranding (blue arrow), and peritoneal cavity (green arrow)

In the ED, the patient received 1 L of normal saline fluid and 20 mEq of potassium. The patient was then transferred to the intensive care unit (ICU) for further monitoring. In the ICU, the patient's altered mental status continued to deteriorate, requiring intubation and mechanical ventilation for airway protection, as the Glasgow Coma Scale was 8. She was then started on normal saline with potassium chloride at 200 cc/hour, an insulin drip, and antibiotics. The patient's blood culture revealed lactobacillus bacteremia, which was treated with broad-spectrum antibiotics. However, she constantly kept spiking a fever with an unidentified source of infection. It was thought the patient could be developing an underlying pneumonia. However, the chest X-ray and CT scan of the chest remained negative. The patient's physical exam revealed severe erythema and induration of the left lower quadrant, with palpation of the overlying skin expressing purulent, foul-smelling, dishwater-like fluid, along with evidence of crepitus. An area of patch dark necrotic tissue approximately 2 × 2 cm was seen at the lateral border of the left lower quadrant, which also expressed a dishwater-like fluid. CT scan abdominal/pelvis revealed subcutaneous emphysema seen at the level of the left lower quadrant of the abdomen extending into the left upper thigh (Figures 1-3). The patient's LRINEC score was 4, indicating a low suspicion for NSTI; however, it does not rule out a diagnosis of NSTI, and further imaging may be required to confirm.

General surgery was consulted, and the patient was transferred to the operating room for debridement. In the operating room, upon incision, necrotic soft tissue was visualized along with foul-smelling dishwater fluid. Necrotic tissue extended down to the external oblique, inferiorly into the lower portion of the thigh, laterally, then medially into the lumbar spine, extending up the spinal column. The wound size was 30 x 30 x 8 cm. Further debridement was deferred due to the extensive involvement of the lumbar spine and hemodynamic instability. The patient was unable to tolerate placement in the prone position, and it was decided to wait 24 hours until the next debridement.

On thorough chart review, the radiology report of the patient's CT lumbar spine taken a month prior during hospitalization at another facility showed extensive postsurgical changes with multiple posterior fusion rods and fixated screws. Unfortunately, the patient's CT imaging could not be obtained from her previous hospital admissions; only the radiologist's report was available, which depicted "fractures of rods of the right lumbar spine at the level of L1 and L4-L5, and on the left at the level of L2 and L5-S1. One of the left fixation rods extends posteriorly and laterally into the soft tissues with a surrounding fluid collection containing gas. There was lucency surrounding the base of the left iliac screw compatible with hardware loosening." Given the patient's presentation and her past surgical history, it was decided to transfer the patient to a tertiary facility for further management. Wound cultures taken during the surgical procedure came back positive for nonhemolytic streptococcus and anaerobic negative rods.

## Discussion

The incidence of spinal hardware infections in adults varies from 0.7% to 20%. However, only a number of studies have reported the development of NSTI secondary to spinal hardware dislodgement [5-7]. NSTIs occur when “flesh-eating bacteria” are introduced into the subcutaneous tissue and begin colonization. Factors that contribute to the increased risk of developing SSI include age, male sex, diabetes, smoking, and obesity [6,7]. In addition, other factors that increase the risk of spinal infections following surgery include operative time of more than five hours, prolonged duration of the spinal implant exposure to air, and prolonged retraction of muscle [6,7]. Particularly, the retraction of the paraspinal muscle often leads to devascularization and a larger dead space, which is conducive for bacterial colonization [6,7].

Infections of spinal implants can be characterized as superficial or deep tissue infections. These two types of infections have different clinical onsets. Superficial tissue infections generally present within the initial two weeks following surgery, accompanied by fever, tenderness, warmth, and purulent drainage [6,7]. Deep tissue infections have a similar onset but a delayed presentation from one month to one year. In addition, bacteria that form biofilms, such as *S. aureus*, have an increased risk of causing SSI and possible NSTI [6,7]. CT scan with contrast remains a vital modality for monitoring spinal hardware positioning as well as any collection of fluid or gas [6,7]. NF can lead to diffuse tissue necrosis, resulting in septic shock and death [7,8].

In this case report, the patient had a past medical history of spinal hardware implants. Two weeks before her admission, an outpatient CT scan of the lumbar spine with contrast done at a neighboring hospital showed a large fluid collection within the left lower back beginning at L2-L3 extending inferiorly into the upper buttocks, containing gas surrounding the displaced hardware rod. Given the patient's clinical presentation, one can concur that the initial site of infection began with the dislodgement of the spinal hardware into the surrounding soft tissue, spreading necrotizing bacteria. Another can propose that the management of this patient, based on her initial CT scan, would be to undergo immediate surgical debridement with broad-spectrum antibiotics and removal of spinal hardware, or a CT-guided fluid aspiration with culture. It is also important to note that the patient's past medical history of uncontrollable type 2 diabetes mellitus could have contributed to her developing an NSTI secondary to spinal hardware malfunction. Evidence has suggested that removal of spinal hardware following infections may increase the risk of postoperative complications such as progression of spinal curvature [9]. The recommended management of infected spinal hardware remains divided regarding removal versus keeping the implant in place and treating conservatively.

During the patient's ICU admissions for severe diabetic ketoacidosis (DKA), her LRINEC was <6, suggesting a relatively low probability of NSTI. However, a low LRINEC score does not rule out the chances of developing NF. Her repeated CT scan showed an extensive accumulation of subcutaneous gas extending from the left back, left flank, and lateral portion of the abdomen, descending into the left thigh, indicating NF. NF spreads along the thoracolumbar fascia, which consists of two anatomical layers, that is, the superficial layer (continuous with the aponeurosis fascia of the latissimus dorsi muscle) and the deep layer (continues toward the fibrotic bands of the lumbar spine) [10]. This intricate system allows for rapid colonization of the fascia. Treatment of NF usually requires multiple rounds of surgical debridement and continuous antibiotics [10].

## Conclusions

The development of NSTI secondary to spinal hardware malfunction is a life-threatening infection. While NSTIs are associated with the infiltration of necrotizing bacteria into the subcutaneous tissue and deep fascia, prosthetic devices implanted into the body carry an increased risk of developing SSI. Bacteria that can form biofilms pose a difficult task to prevent infections. Early recognition of NSTI is crucial for providing effective surgical management and preventing excessive loss of vital tissue. Patients with concerning comorbidities should be followed carefully to rule out the development of NSTI in a critical care setting. Ordering additional imaging on critically ill patients with known comorbidities or spinal hardware malfunction could be the first step in diagnosing the formation of NSTI. Patients with NSTI should be aggressively treated with multiple rounds of surgical debridement and broad-spectrum antibiotics.
